# Integrated Flavoromics Reveals Breed-Associated Flavor Evolution in Dry-Cured Xuanwei Ham During Ripening

**DOI:** 10.3390/foods15203592

**Published:** 2026-10-10

**Authors:** Jialin Li, Hui Liu, Zhiqing Tian, Chang Wang, Wusun Li, Xiaoyan Tang

**Affiliations:** 1 State Key Laboratory for Quality and Safety of Agro-Products, Institute of Quality Standards and Testing Technology for Agro-Products, Chinese Academy of Agricultural Sciences, Beijing 100081, China; 17793650420@163.com (J.L.); liuhui02@caas.cn (H.L.); zqtian1996@163.com (Z.T.); 15552155502@163.com (C.W.); liwusun@caas.cn (W.L.); 2 Key Laboratory of Agro-Product Quality and Safety, Ministry of Agriculture and Rural Affairs, Beijing 100081, China

**Keywords:** Xuanwei ham, pig breed, flavor metabolites, gas chromatography ion mobility, discriminant markers

## Abstract

Xuanwei ham (XWH), a Protected Geographical Indication (PGI) dry-cured ham traditionally produced from indigenous Xuanhe pigs, is increasingly threatened by substitution with lower-cost hams made from commercial Duroc × Landrace × Yorkshire (DLY) pigs. This study compared XWH- and DLY-derived ham (DLYH) at 1, 2, and 3 years of ripening using an electronic tongue (E-tongue), an electronic nose (E-nose), gas chromatography-ion mobility spectrometry (GC-IMS), and targeted analysis of nucleotides, free amino acids (FAAs), and free fatty acids (FFAs). The results showed that XWH exhibited stronger umami and greater proteolysis, accompanied by greater accumulation of glutamic acid, whereas DLYH showed greater bitterness. After three years of ripening, XWH contained higher total FFAs, oleic acid, and linoleic acid and maintained a more stable UFA/SFA ratio than DLYH. GC-IMS showed prominent 2,5-dimethylfuran and 2-methylpyrazine in XWH, whereas lipid-oxidation-related 2-heptanone and 1-octen-3-ol were more prominent in DLYH. Partial Least Squares Discriminant Analysis (PLS-DA) clearly separated the six ham groups and identified 30 key discriminant markers. These findings reveal distinct breed-associated flavor evolution during ripening and provide potential chemical markers for the quality evaluation and authentication of XWH.

## 1. Introduction

Xuanwei ham (XWH), one of the most representative traditional dry-cured hams in China, is highly appreciated for its unique and complex flavor profile. The distinctive sensory attributes of authentic XWH are closely associated with its characteristic raw material, derived from indigenous Xuanhe pigs native to the Xuanwei region of Yunnan Province [1,2]. Compared with commercial crossbred pigs, particularly Duroc × Landrace × Yorkshire (DLY), Xuanhe pigs possess favorable meat-quality traits, including relatively high intramuscular fat content [3], which can provide abundant lipid precursors for the generation of flavor compounds during ham ripening and aging [4]. Owing to the considerable price premium of genuine XWH, economically motivated adulteration has emerged as a serious concern. In practice, hams manufactured from DLY pigs may be mislabeled and sold as authentic XWH. Such raw-material substitution can compromise product quality, mislead consumers, disrupt fair trade, and undermine the protected geographical indication (PGI) status of the product. Therefore, the identification of reliable flavor markers that can objectively discriminate genuine XWH from adulterated products has become a priority for quality control and market surveillance.

The flavor of dry-cured ham is shaped by the accumulation and transformation of small-molecule metabolites during ripening through endogenous enzymatic reactions, microbial metabolism, and non-enzymatic processes [5]. Among these metabolites, nucleotides, free amino acids (FAAs), small peptides, and free fatty acids (FFAs) are particularly important because they contribute directly to taste and also serve as precursors for aroma formation. FAAs and peptides affect umami, sweetness, bitterness, and aftertaste, while participating in Maillard and Strecker reactions that generate aroma-active compounds [6,7]. Lipid hydrolysis releases FFAs, especially unsaturated FFAs, which can undergo further oxidative degradation to generate aldehydes, ketones, alcohols, alkylfurans, and other volatile compounds, thereby contributing pronounced fatty, bean-like, and aged oxidative aroma notes [8,9]. The formation and transformation of these compounds depend not only on ripening time but also on breed-related properties of the raw material. Indigenous and commercial pig breeds differ in intramuscular fat deposition, fatty acid composition, endogenous proteolytic and lipolytic activities, and antioxidant capacity, thereby providing distinct initial pools of flavor precursors and potentially different metabolic trajectories during curing and ripening [10,11,12].

Recent studies have increasingly combined biomimetic sensory analysis with chemical characterization to describe the flavor attributes of meat products and elucidate their underlying chemical basis. Electronic tongue (E-tongue) analysis rapidly captures overall taste patterns, including variation in sourness, saltiness, umami, sweetness, and bitterness [13]. Electronic nose (E-nose) analysis, based on cross-reactive sensor arrays, offers a rapid and non-destructive characterization of overall odor profiles and has been applied to classify dry-cured ham samples according to processing conditions, geographical origin, breed, and feeding regime [14,15,16]. Gas chromatography-ion mobility spectrometry (GC-IMS) separates and detects volatile organic compounds (VOCs) according to their chromatographic retention and ion-mobility characteristics, generating visual fingerprints that facilitate comparisons of volatile composition among samples [17,18,19]. In parallel, chromatographic techniques, including chromatography–mass spectrometry, have been widely used to identify and quantify non-volatile taste-active compounds, thereby providing biochemical evidence for flavor development and ripening-related changes [20,21]. Previous studies on XWH have described changes in physicochemical properties, microbial communities, water-soluble metabolites, FFAs, and VOCs during processing and ripening [1,22,23]. However, direct comparisons of flavor evolution between XWH produced from indigenous Xuanhe pigs and ham produced from commercial DLY pigs under the same processing conditions remain limited, particularly across different ripening stages. Therefore, how pig breed influences the evolution of flavor-related compounds during long-term ripening, and which compounds may serve as potential discriminant markers, remain insufficiently understood.

To address these knowledge gaps, XWH- and DLY-derived ham (DLYH) were compared over 1–3 years of ripening. The objectives were to: (1) rapidly characterize flavor differences between the two types of ham using biomimetic sensory technologies, including E-tongue and E-nose; (2) construct volatile fingerprints using GC-IMS; (3) integrate these fingerprints with targeted profiling of flavor-related metabolites, including nucleotides, FAAs, and FFAs, to elucidate the chemical basis of breed-driven flavor differentiation; and (4) screen candidate discriminant markers for XWH authentication using multivariate statistical analysis. By comparing the two ham types across multiple ripening stages, this study provides new insights into breed-associated flavor evolution and offers a chemical basis for the quality evaluation and authenticity screening of XWH.

## 2. Materials and Methods

### 2.1. Materials

The hams (XWH and DLYH) used in this study were provided by Yunnan Puji Ham Food Co., Ltd. (Xuanwei, China). Both Xuanhe and DLY pigs were reared under intensive farming conditions and fed the same commercial diet, consisting of corn, soybean meal, wheat bran, soybean oil, L-lysine, limestone, and calcium phosphate. All breeding and slaughtering procedures complied with relevant animal welfare and ethical standards. Hind legs were obtained from pigs of comparable age and body weight within the same production batch. Both types of ham were processed according to the XWH Production Technical Specification (GB/T 18357-2008) [24], including green-ham preparation, trimming and shaping, blood-stasis squeezing, salting, stacking and turning, washing and shaping, air drying, fermentation, and ripening. Processing was conducted from January 2023 to January 2026. During this period, six hams from each ham type were randomly selected annually in January. The biceps femoris muscles were excised, trimmed of visible fat, minced, vacuum-packaged, and stored at −20 °C until analysis. XWH samples from the first, second, and third ripening years were named XWH1, XWH2, and XWH3, respectively, whereas DLYH samples were named DLYH1, DLYH2, and DLYH3. The basic physicochemical properties of both ham types are presented in Table 1.

### 2.2. Chemicals

Cytidine 5′-monophosphate (5′-CMP), uridine 5′-monophosphate (5′-UMP), adenosine 5′-monophosphate (5′-AMP), guanosine 5′-monophosphate (5′-GMP), inosine 5′-monophosphate (5′-IMP), xanthosine 5′-monophosphate (5′-XMP), hypoxanthine (Hx), and inosine (HxR) were purchased from Sigma-Aldrich (St. Louis, MO, USA). A 37-component fatty acid methyl ester (FAME) standard mixture (C4–C24) was purchased from Anpu Experimental Technology Co., Ltd. (Shanghai, China). Trichloroacetic acid, sodium hydroxide, n-hexane, and sulfosalicylic acid were purchased from Macklin Biochemical Technology Co., Ltd. (Shanghai, China). Triethylamine and methanol were purchased from Merck (Darmstadt, Germany) and were of HPLC grade. A 17-component amino acid standard mixture was purchased from Solarbio (Beijing, China), while amino acid analysis buffers (PH series) and ninhydrin reagent were purchased from Hitachi (Tokyo, Japan).

### 2.3. E-Tongue Analysis

The taste characteristics of ham samples were objectively evaluated using an Astree E-tongue system (Alpha M.O.S., Toulouse, France) equipped with seven potentiometric biomimetic taste sensors and an Ag/AgCl reference electrode. Five sensors, namely AHS (sourness), CTS (saltiness), NMS (umami), ANS (sweetness), and SCS (bitterness), were used to acquire basic taste information, whereas PKS and CPS were used as composite cross-selective sensors for the overall taste profile.

A 6 g portion of minced ham was homogenized with 30 mL of ultrapure water using a high-speed disperser (IKA, Staufen, Germany) at 10,000 rpm for 30 s per cycle over three cycles. The homogenate was incubated at 40 °C for 1 h with agitation. After filtration, the supernatant was centrifuged (10,000 rpm, 15 min, 4 °C), refrigerated at 4 °C for 12 h to remove solidified fat, and centrifuged again (10,000 rpm, 10 min, 4 °C) to obtain the test solution.

For E-tongue analysis, the test solution was diluted 15-fold with ultrapure water, and 25 mL of the diluted solution was transferred to a dedicated beaker. Before measurement, the sensors and electrode were activated, and the instrument was calibrated. Each sample was analyzed in six technical replicates, and the three most stable responses were retained for statistical analysis.

### 2.4. FAA Analysis

A 1 g portion of minced ham sample was homogenized with 15 mL of 0.02 mol/L HCl (GR) using a high-speed disperser (IKA, Staufen, Germany) at 12,000 rpm for 30 s, followed by ultrasonic extraction for 5 min and centrifugation at 10,000 rpm for 10 min at 4 °C. The supernatant was collected, and the residue was re-extracted under the same conditions. The combined supernatants were diluted to a final volume of 50 mL with ultrapure water. Subsequently, 4 mL of the extract was mixed with 4 mL of 4% sulfosalicylic acid, allowed to stand for 20 min, and centrifuged at 11,000 rpm for 10 min at 4 °C. The resulting supernatant was filtered through a 0.22 μm aqueous membrane filter.

FAAs were analyzed using an automatic amino acid analyzer (L-8900, Hitachi, Japan) equipped with a cation-exchange analytical column (60 × 4.6 mm, 3 μm) and a post-column derivatization reaction column (40 × 4.6 mm). Amino acids were separated using sodium citrate buffers at a flow rate of 0.35 mL/min, followed by post-column derivatization with ninhydrin at a flow rate of 0.30 mL/min and detection at 440 and 570 nm. A mixed amino acid standard solution (100 nmol/mL) was used for identification based on retention times and quantification by a single-point external standard method using the corresponding sample-to-standard peak-area ratios.

### 2.5. Analysis of 5′-Nucleotides and Related Metabolites

A 3 g portion of minced ham sample was weighed into a centrifuge tube, mixed with 10 mL of pre-cooled 5% trichloroacetic acid solution by vortexing, ultrasonicated for 15 min, and centrifuged (10,000 rpm, 10 min). The supernatant was collected, and the residual pellet was re-extracted using the same procedure. The supernatants were combined, adjusted to pH 6.5 with 3 mol/L NaOH, made up to a volume of 50 mL with ultrapure water, vortexed, and filtered through a 0.22 μm hydrophilic membrane filter. The filtrate was transferred to 2 mL vials for HPLC analysis and quantified using external standards.

Chromatographic conditions: ZORBAX SB-C18 column (250 × 4.6 mm, 5 μm; Agilent, Santa Clara, CA, USA), 30 °C; mobile phase: methanol: 1% triethylamine aqueous solution (pH 3.0, phosphoric acid-adjusted) (3:97, *v*/*v*); flow rate: 0.8 mL/min; injection volume: 10 μL; detection wavelength: 254 nm.

### 2.6. FFA Analysis

Lipid extraction and derivatization were performed as described by Zhu et al. [25], with minor modifications. A 5 g sample was mixed with 25 mL n-hexane, ultrasonicated for 20 min, allowed to stand for 2 h, and centrifuged (3500 rpm, 4 °C, 15 min). The supernatant was evaporated to constant weight under nitrogen (44 °C) to determine total lipid content. For derivatization, 20 mg of lipid was dissolved in 2 mL of n-hexane, mixed with 400 μL of 1 mol/L KOH-methanol, vortexed for 5 min, treated with anhydrous sodium sulfate (shaken for 2 min), and phase-separated. The supernatant was filtered (0.22 μm organic membrane) and transferred to 2 mL vials. FFAs were quantified using a 37-component FAME external standard mixture.

GC conditions: separation was performed on a J&W Select FAME column (100 m × 0.25 mm × 0.25 μm; Agilent, Santa Clara, CA, USA) with injector and detector temperatures set to 250 °C. High-purity helium was used as the carrier gas at a constant flow rate of 3 mL/min. The injection volume was 1 μL with a split ratio of 50:1. The temperature program was as follows: 120 °C held for 1 min, ramped to 160 °C at 20 °C/min, further ramped to 166 °C at 1 °C/min and held for 1 min, then ramped to 198 °C at 0.5 °C/min and held for 2 min, and finally ramped to 230 °C at 5 °C/min and held for 10 min.

### 2.7. E-Nose Analysis

The odor profiles of ham samples were characterized using a Heracles II E-nose system (Alpha M.O.S., Toulouse, France), with slight modifications to the method described by Tian et al. [26]. A 2 g sample was incubated in a 20 mL headspace vial at 50 °C for 10 min with stirring at 500 rpm. Hydrogen was used as the carrier gas at a flow rate of 1 mL/min. The injection volume was 1000 μL, and the injector temperature was 200 °C. The oven was held at 60 °C for 2 min, ramped to 270 °C at 3 °C/s, and held for 20 s.

### 2.8. GC-IMS Analysis

VOCs in ham samples at different ripening stages were analyzed using GC-IMS. A 2 g sample was weighed into a 20 mL headspace vial and incubated at 60 °C for 20 min. VOC spectra were acquired and processed using LAV 2.2.1 software, and compounds were identified by comparison with the integrated NIST 2017 and IMS databases. C4–C9 ketones were used as external standards for retention index (RI) calibration.

GC conditions: isothermal separation was performed on an MXT-5 column (Restek, Bellefonte, PA, USA) maintained at 60 °C. High-purity nitrogen (≥99.999%) was used as the carrier gas, with the flow rate programmed as follows: 2 mL/min for 0–2 min, 10 mL/min for 2–10 min, 100 mL/min for 10–20 min, and 150 mL/min for 20–30 min, giving a total run time of 30 min. IMS conditions: a ^3^H radioactive ionization source (300 MBq) was used; the drift tube was set at 9.8 cm in length, with a temperature of 45 °C and a voltage of 5000 V; and high-purity nitrogen was used as the drift gas at a constant flow rate of 150 mL/min for 30 min.

### 2.9. Evaluation of Key Flavor Compounds

#### 2.9.1. Relative Odor Activity Value (ROAV)

The ROAVs of VOCs were calculated following the method of Ma et al. [27]. The compound with the highest ratio of relative abundance to odor threshold was assigned an ROAV of 100, and the ROAVs of the remaining compounds were calculated as follows:

$${\mathrm{ROAV}}_{i}=\frac{C_{i}}{C_{\mathrm{ref}}}\times \frac{T_{\mathrm{ref}}}{T_{i}}\times 100$$

C_i_ and T_i_ represent the relative abundance and odor threshold of compound (i), respectively, while C_ref_ and T_ref_ are the corresponding values for the reference compound. Compounds with ROAV ≥ 1 were considered key contributors to ham aroma, whereas those with 0.1 ≤ ROAV < 1 were regarded as potential aroma modifiers.

#### 2.9.2. Taste Activity Values (TAVs)

TAVs were calculated according to Wang et al. [28] to estimate the potential contributions of individual taste-active compounds to ham taste:

$${\mathrm{TAV}}_{i}=\frac{C_{i}}{T_{i}}$$

C_i_ and T_i_ represent the concentration and taste threshold of compound (i), respectively. Compounds with TAV ≥ 1 were considered potential key contributors to ham taste.

### 2.10. Statistical Analysis

Excel 2021 was used for data organization, and statistical analyses were performed using SPSS 20.0 (IBM Corp., Armonk, NY, USA). Each group included six biological replicates, with each measurement performed in triplicate. Data are expressed as mean ± SD. The effects of ham type, ripening time, and their interaction were analyzed using two-way ANOVA, followed by Tukey’s multiple-comparison test when appropriate. Differences were considered statistically significant at *p* < 0.05. Multivariate analyses were conducted using SIMCA 14.1, and figures were generated using the online Chiplot platform (https://chiplot.online/).

## 3. Results and Discussion

### 3.1. E-Tongue Analysis of Taste Differences Between XWH and DLYH During Ripening

**Figure 1:**
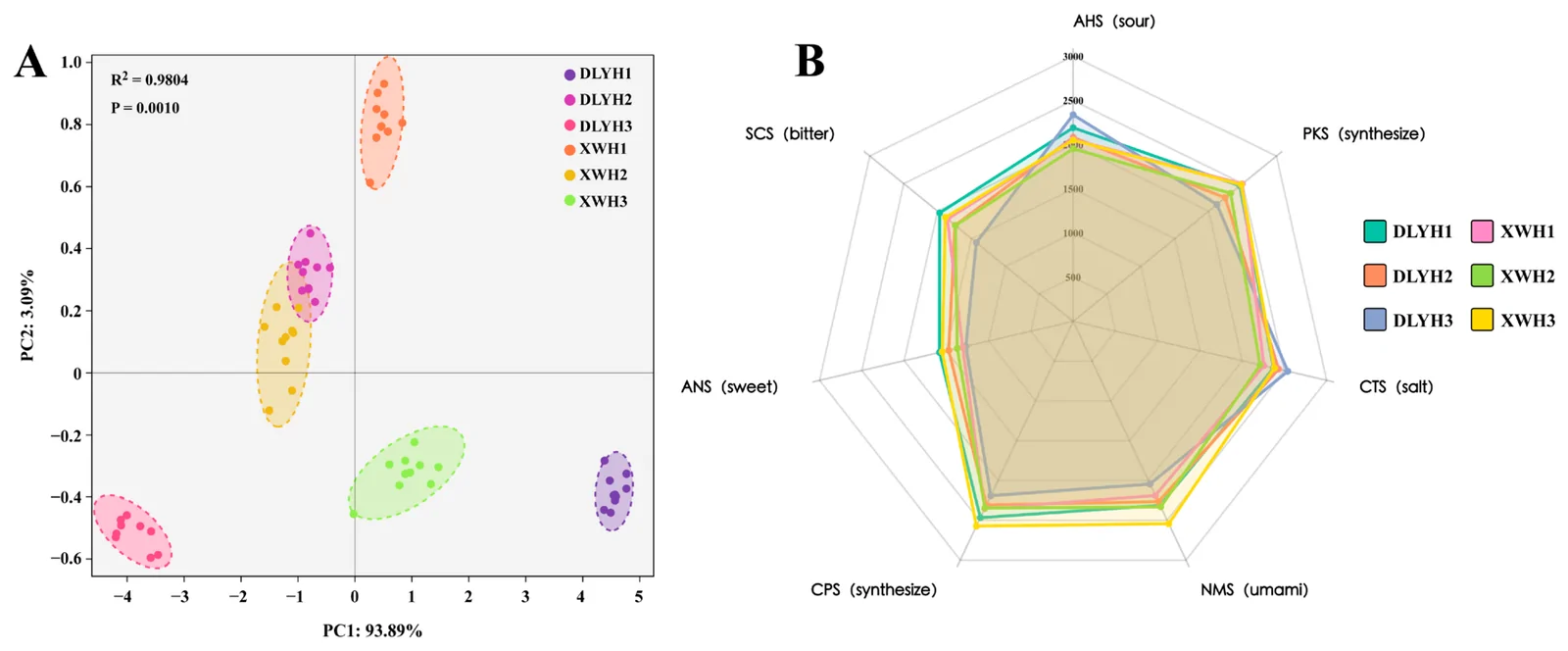
E-tongue analysis of taste profiles in different ham samples. (**A**) PCA score plot; (**B**) taste radar chart.

The E-tongue employs a multisensor array with biomimetic lipid-sensitive membranes to respond to taste-related compounds and characterize the overall taste profile of samples [13]. Principal component analysis (PCA) of the E-tongue data showed that PC1 and PC2 explained 96.98% of the cumulative variance (Figure 1A). The samples were distributed discretely without overlap, demonstrating the excellent discriminatory capacity of the E-tongue for taste differences among ham samples. The radar chart (Figure 1B) revealed significant differences in taste attributes, mainly characterized by umami, saltiness, and sourness. XWH samples, especially those from the second and third ripening years, exhibited significantly higher umami-related responses than their DLYH counterparts (*p* < 0.05). In contrast, DLYH samples showed higher bitterness- and sourness-related responses. These results indicate distinct taste-related response patterns between XWH and DLYH during ripening. Similarly, Wen et al. [2] found that pig breed, including Landrace, Wujin, and Tibetan fragrant pigs, significantly influenced the quality and flavor-related characteristics of ham, with umami-related compounds being important determinants of ham flavor quality.

### 3.2. Differences in Small-Molecule Metabolites Between XWH and DLYH During Ripening

#### 3.2.1. Analysis of FAAs

FAAs are important taste-active compounds and aroma precursors in meat products, and their composition and abundance substantially influence flavor development during ripening. Analysis of FAA composition across the six ham groups revealed distinct FAA profiles between XWH and DLYH (Table 2). A total of 17 FAAs were detected. Bitter and sweet amino acids were the predominant taste-related FAA categories, accounting for 34.02–45.03% and 36.69–44.08% of total FAAs, respectively, followed by umami amino acids, which accounted for 15.40–21.67%. The four most abundant amino acids were glutamic acid (Glu, 4.319–9.444 mg/g), lysine (Lys, 3.795–7.795 mg/g), alanine (Ala, 2.999–6.185 mg/g), and leucine (Leu, 2.635–4.694 mg/g), which together accounted for nearly half of the total FAAs. This distribution is broadly consistent with that reported by Tao et al. [29], who found that Glu, Ala, Lys, and Arg were the predominant amino acids in Diannan small-ear pig ham. High levels of Glu and Ala contribute to the umami and sweet tastes of ham [30]. During ripening, the total FAA content increased from 33.242 mg/g in XWH1 to 60.727 mg/g in XWH3, whereas the corresponding increase in DLYH was from 37.793 mg/g in DLYH1 to 46.007 mg/g in DLYH3. At the final ripening stage, the total FAA content was significantly higher in XWH3 than in DLYH3 (*p* < 0.05), indicating more extensive accumulation of FAAs in XWH during ripening. Previous studies have shown that endogenous proteolytic enzymes, including cathepsins and aminopeptidases, participate in FAA formation during the processing of dry-cured meat products [10,31]. In addition to endogenous proteolysis, microbial metabolism may also contribute to FAA accumulation. Zhou et al. [32] reported that differential metabolites between Xuanwei and Iberian hams were mainly enriched in amino acid metabolism pathways, with dominant microorganisms such as Staphylococcus and Debaryomyces closely associated with these metabolic differences. Therefore, the distinct FAA accumulation patterns between XWH and DLYH may reflect the combined effects of breed-related muscle characteristics, endogenous proteolytic activity, and microbial metabolism during ripening. As a key umami compound, Glu reached its highest concentration in XWH3 (9.44 mg/g), approximately 1.5 times that detected in DLYH3. In addition, the ratio of sweet- and umami-tasting amino acids to bitter-tasting amino acids was generally higher in XWH (1.43–1.93) than in DLYH (1.21–1.36). This compositional pattern suggests a greater relative contribution of desirable taste-active amino acids in XWH, which may contribute to a more balanced taste profile. These findings are consistent with the E-tongue results, which showed stronger umami and a more favorable taste balance in XWH.

#### 3.2.2. Analysis of 5′-Nucleotides and Their Metabolites

**Figure 2:**
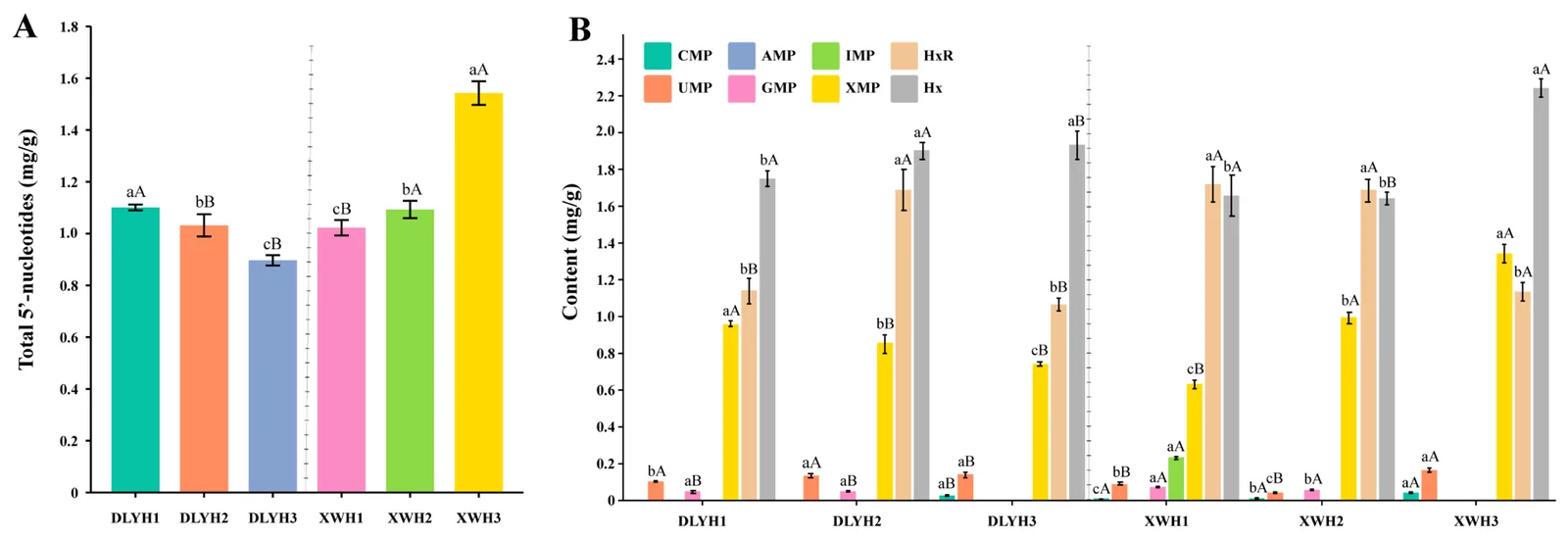
Profiles of 5′-nucleotides and related metabolites in different ham samples. (**A**) Total 5′-nucleotides; (**B**) individual 5′-nucleotides and related metabolites. Different lowercase letters indicate significant differences among ripening times within the same breed, whereas different uppercase letters indicate significant differences between breeds at the same ripening time (*p* < 0.05).

In food flavor chemistry, 5′-nucleotides are important taste-active compounds mainly generated by enzymatic hydrolysis of nucleic acids [34,35]. These nucleotides enhance sweet and meaty perceptions, suppress salty, sour, and bitter tastes, and exhibit a strong synergistic effect with Glu and Asp by lowering the umami detection threshold [36]. As shown in Figure 2, the total 5′-nucleotides declined continuously in DLYH, whereas XWH showed a different temporal pattern. 5′-AMP was not detected in any sample. As an important umami-enhancing nucleotide in meat and meat products, 5′-IMP was detected only in XWH1 and was not detected in the remaining samples. Meanwhile, both ham types accumulated HxR (1.067–1.722 mg/g) and Hx (1.643–2.246 mg/g), which are downstream products of 5′-IMP degradation. The accumulation of HxR and Hx therefore supports the progressive degradation of 5′-IMP during ripening, consistent with previous findings in long-ripened hams [21]. Hx may contribute to bitterness [37]. Although Hx levels were comparable between the two ham types, its contribution to the overall bitterness-related response may depend on the composition of other taste-active compounds present in the samples. In the present study, XWH contained higher levels of umami-active amino acids, whereas DLYH showed lower levels of these compounds. This difference in the overall taste composition may partly contribute to the different bitterness-related responses observed between the two ham types. Thus, although nucleotide degradation generally followed a similar pathway in the two ham types, differences in the overall composition of taste-active compounds may have influenced their taste-related response patterns.

#### 3.2.3. Analysis of FFAs

FFAs serve as key substrates for lipid oxidation and the formation of volatile aroma compounds in dry-cured ham [38]. In this study, 19 FFAs were detected in the two ham types, including 8 saturated fatty acids (SFAs), 6 monounsaturated fatty acids (MUFAs), and 5 polyunsaturated fatty acids (PUFAs) (Table 3). Total FFA content increased continuously during the three-year ripening period, confirming progressive lipolysis. At the final ripening stage, the total FFA content reached 12.596 g/100 g in XWH3, which was significantly higher than that in DLYH3 (11.487 g/100 g, *p* < 0.05), indicating greater FFA accumulation and a larger pool of lipid-derived aroma precursors in XWH.

Long-chain fatty acids predominated in the FFA profiles of both ham types, with oleic acid, palmitic acid, linoleic acid, and stearic acid being the major components. Oleic acid was the most abundant FFA and increased markedly during ripening. XWH consistently showed higher oleic acid levels than DLYH at corresponding ripening stages, reaching 5.469 g/100 g in XWH3, compared with 5.066 g/100 g in DLYH3. As an important precursor of lipid-oxidation-derived aldehydes, ketones, and alcohols, oleic acid, the greater accumulation of oleic acid in XWH may contribute to differences in lipid-derived aroma formation between the two ham types [39]. Palmitic acid increased sharply in both ham types, reaching 3.200 g/100 g in XWH3 and 3.085 g/100 g in DLYH3, with no significant difference between the two groups (*p* > 0.05), suggesting that long-term ripening reduced differences in major SFA release. Linoleic acid also showed clear breed-related differences, increasing from 1.212 to 1.952 g/100 g in XWH and from 1.196 to 1.540 g/100 g in DLYH. As an oxidation-prone PUFA, linoleic acid is an important precursor of volatile aldehydes and furans that impart green, fatty, and roasted notes to dry-cured ham [4]. More broadly, the contribution of breed-dependent fatty acid profiles to pork flavor has also been demonstrated by Wu et al. [40], who, through multivariate statistical analysis, identified oleic acid, docosahexaenoic acid, and α-linolenic acid as major fatty acid precursors associated with the rich flavor of Chinese indigenous pig breeds by comparing Beijing Black pigs and Laiwu pigs with commercial DLY pigs.

The UFA/SFA ratio further reflected differences in the relative balance between unsaturated and saturated fatty acids in the two ham types. This ratio decreased markedly in DLYH from 1.971 to 1.620, whereas it remained relatively stable in XWH, ranging from 1.793 to 1.909. The more pronounced decrease in DLYH indicates that the accumulation of UFAs was lower relative to that of SFAs during ripening, which may reflect breed-dependent differences in lipid composition and transformation [23]. In addition, several FFAs, such as palmitoleic acid, myristic acid, (Z)-11-eicosenoic acid, and erucic acid, were generally more abundant in XWH, further reflecting breed-dependent lipid fingerprints.

### 3.3. Differences in VOCs Between XWH and DLYH During Ripening

#### 3.3.1. Analysis of E-Nose

**Figure 3:**
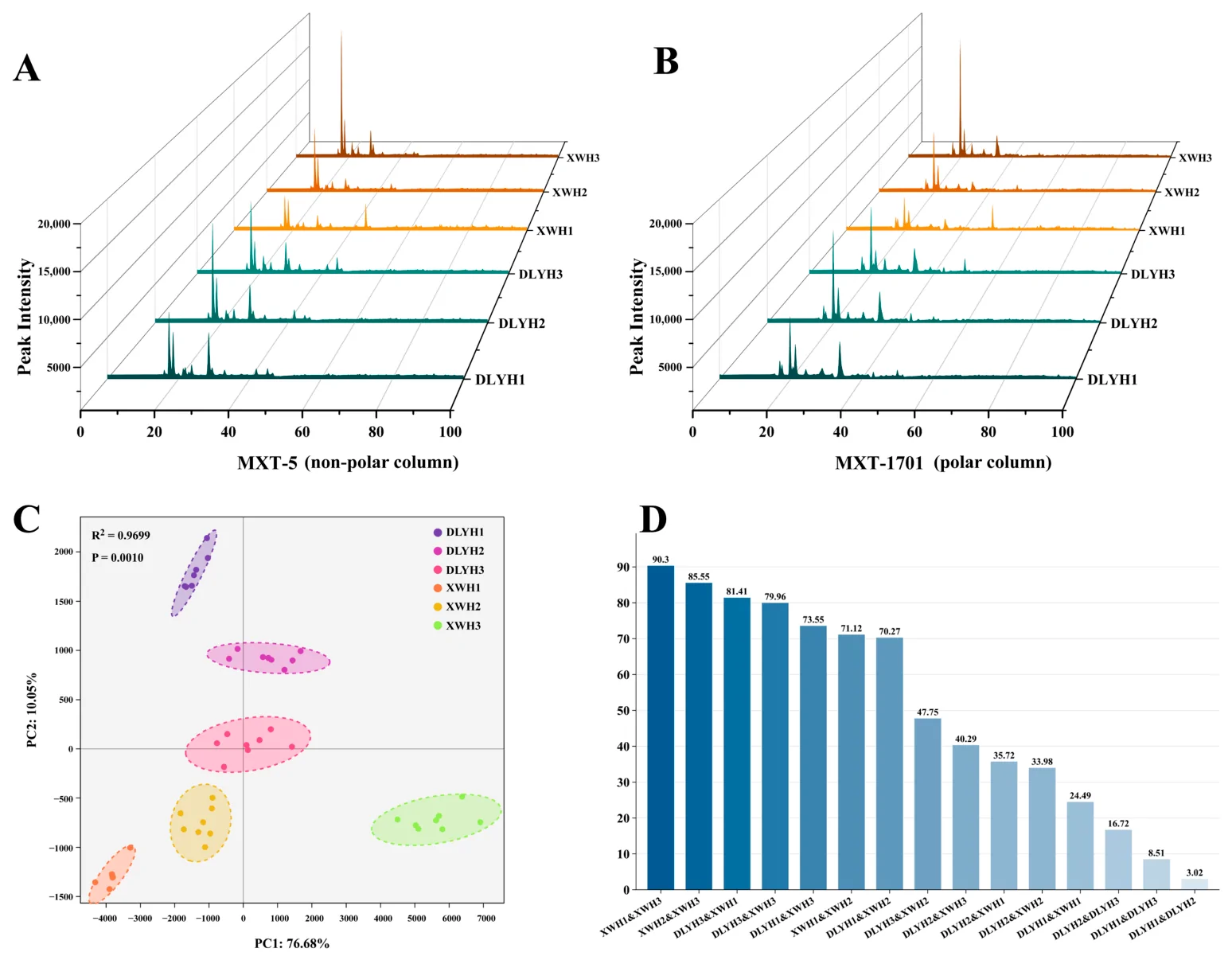
E-nose analysis of volatile profiles in different ham samples. (**A**,**B**) Chromatographic fingerprints; (**C**) PCA score plot; (**D**) Pairwise DI among different ham samples.

VOC profiles of ham samples were characterized using E-nose analysis. The system was equipped with an MXT-5 non-polar column and an MXT-1701 polar column, allowing comprehensive separation and detection of VOCs through the complementary selectivity of the two stationary phases. The chromatographic fingerprints obtained from the non-polar and polar columns are shown in Figure 3A and B, respectively, for XWH and DLYH samples at different ripening stages. Multiple characteristic peaks were detected in all samples, while clear differences in peak number, intensity, and distribution were observed among the different ham samples.

PCA of the E-nose data showed that PC1 and PC2 together explained 86.73% of the total variance, indicating that the major variation in the original volatile fingerprint data was well represented (Figure 3C). The different ham samples exhibited clear separation in the score plot. Notably, XWH samples were mainly distributed along PC1, whereas DLYH samples showed a greater degree of dispersion along PC2, suggesting distinct breed-dependent volatile profiles.

To further quantify the differences in volatile fingerprints among the six ham samples, pairwise discrimination index (DI) analysis was performed (Figure 3D). The highest DI values were observed for XWH1 vs. XWH3 and XWH2 vs. XWH3, reaching 90.30% and 85.55%, respectively, indicating pronounced changes in the volatile composition of XWH during ripening. The DI value between DLYH3 and XWH1 was also high at 81.41%, reflecting a marked difference between the two breeds. In contrast, the overall VOC fingerprints of DLYH remained relatively stable across ripening stages, with DI values of 16.72%, 8.51%, and 3.02% for DLYH2 vs. DLYH3, DLYH1 vs. DLYH3, and DLYH1 vs. DLYH2, respectively. These results indicate that pig breed is an important factor shaping the VOC fingerprint of ham, while the ripening process further amplifies breed-related differences in VOCs.

#### 3.3.2. Analysis of GC-IMS

**Figure 4:**
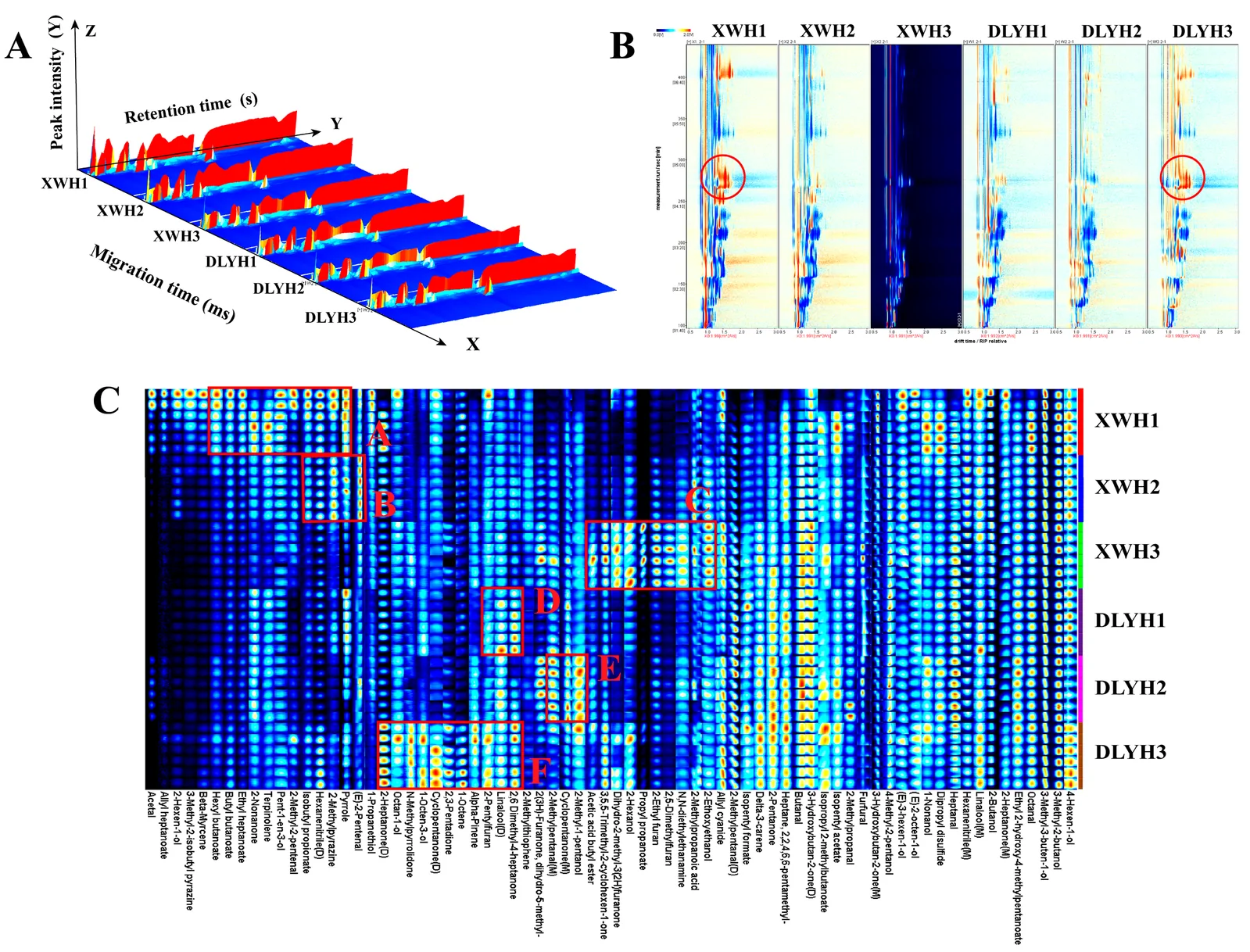
GC-IMS profiles of VOCs in different ham samples. (**A**) 3D topographic map; (**B**) 2D difference map (reference: XWH3); (**C**) VOC fingerprint. Rows represent samples, columns represent VOCs, and color intensity indicates relative content.

The composition and relative abundance of VOCs are directly associated with the flavor profiles and sensory quality of ham [25]. In this study, GC–IMS was employed as a rapid visual fingerprinting technique, and 72 VOCs were tentatively identified across the six ham groups (Figure 4). These compounds comprised 17 alcohols, 14 ketones, 11 esters, 10 aldehydes, 1 acid, 6 terpenes, 1 hydrocarbon, and 12 compounds from other chemical classes (Table S1). Based on the summed signal intensity, alcohols represented the dominant chemical class, accounting for 31.22–40.96% of the total VOC signal.

The three-dimensional topographic map (Figure 4A), based on retention time, drift time, and peak intensity, visualized the overall VOC distribution among samples. In the two-dimensional difference maps (Figure 4B), using the reaction ion peak as the reference, each data point represents a VOC, and color intensity indicates signal intensity (red, strong; white, weak) [41]. Within the region corresponding to retention times of approximately 250–300 s and drift times of 1.2–1.7 ms, XWH1 and DLYH3 exhibited stronger VOC signals than XWH3. These results indicate that VOC composition and signal intensity differed by breed and ripening time.

Figure 4C further presents VOC fingerprints, in which some compounds showed monomer (M) and dimer (D) signals caused by association of ions with neutral molecules [42]. Octanal, heptanal, 4-hexen-1-ol, 3-methyl-3-buten-1-ol, furfural, butanal, and 3-methyl-2-butanol, among other compounds, were detected in all ham samples. These common VOCs contributed to the characteristic fatty, malty, and sweet aroma background of the hams. XWH exhibited distinct stage-specific VOC patterns, with several heterocyclic compounds becoming prominent during ripening. XWH1 (region A) showed relatively strong signals for hexyl butanoate, butyl butanoate, ethyl heptanoate, 2-nonanone, terpinolene, and 1-penten-3-ol. In XWH2 (region B), 2-methylpyrazine, pyrrole, and (E)-2-pentenal became more prominent, whereas XWH3 (region C) displayed pronounced responses for 2-ethylfuran, 2,5-dimethylfuran, 2-ethoxyethanol, 2-hexanol, and dihydro-2-methyl-3(2H)-furanone. Pyrazines are generally formed via Strecker degradation, in which α-dicarbonyl compounds react with amino acids to generate α-aminoketone intermediates that subsequently undergo condensation and oxidation to form the pyrazine ring, whereas furans may arise from sugar degradation and lipid oxidation [43]. The higher levels of FAAs in XWH may therefore have provided more amino precursors for these reactions, contributing to the formation of desirable roasted, caramel-like, and sweet aroma notes [44]. In contrast, DLYH showed relatively stronger responses for several VOCs associated with lipid oxidation, including aldehydes, ketones, alcohols, and hydrocarbons. DLYH1 (region D) exhibited prominent signals for 2,6-dimethylheptan-4-one and linalool (D), while 2-methyl-1-pentanol, 2-methylpentanal (M), and cyclopentanone (M) were more pronounced in DLYH2 (region E). In DLYH3 (region F), relatively strong responses were observed for 2-heptanone (D), 1-octanol, N-methylpyrrolidone, 1-octen-3-ol, cyclopentanone (D), 2,3-pentanedione, and 1-octene.

### 3.4. Multivariate Statistical Analysis and Identification of Discriminant Characteristic Markers

**Figure 5:**
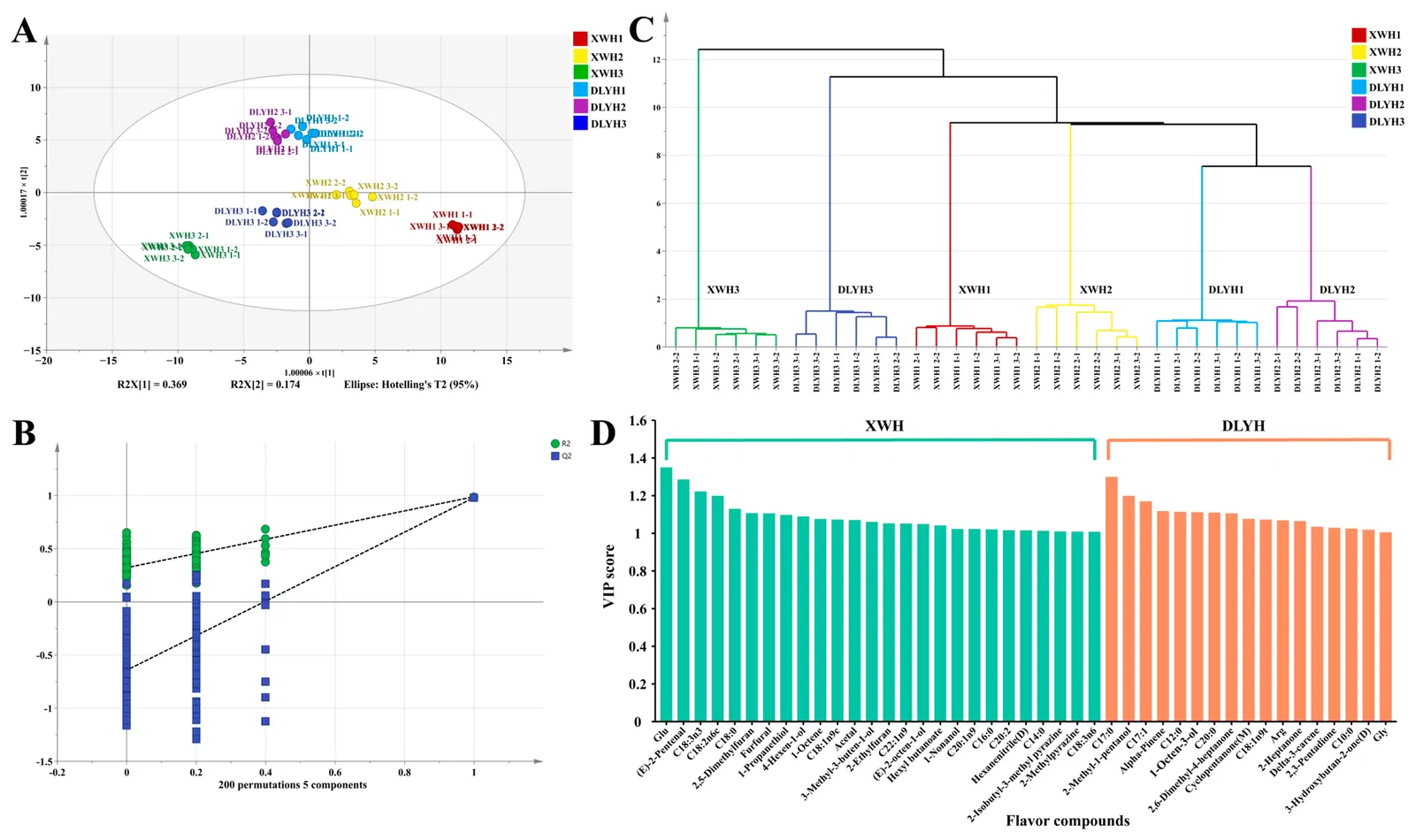
PLS-DA of characteristic flavor compounds in different ham samples. (**A**) PLS-DA score plot; (**B**) 200-permutation test; (**C**) HCA clustering diagram; (**D**) VIP value.

PLS-DA was applied to the combined dataset of these flavor-related indicators to evaluate the separation among the six ham groups and to screen for potential discriminant markers. As shown in Figure 5, the model exhibited good fit and predictive ability, with R^2^X = 0.918, R^2^Y = 0.981, and Q^2^ = 0.961 (>0.5). The 200-permutation test yielded a Q^2^ intercept of −0.657 (<0) (Figure 5B), indicating that the model was not overfitted and was reliable for discriminating the sample groups investigated in this study [45]. The score plot showed clear separation among all six groups (Figure 5A). DLYH1 and DLYH2 clustered closely, indicating relatively similar flavor characteristics during the early ripening stage of DLYH, whereas the three XWH groups were more dispersed, indicating more pronounced ripening-associated changes in XWH. As shown in Figure 5C, hierarchical cluster analysis (HCA) further classified the samples into five clusters [46]. Group I (DLYH1, DLYH2), Group II (XWH2), Group III (XWH1), Group IV (DLYH3), and Group V (XWH3). The independent clustering of XWH3 further indicates its distinctive and complex flavor profile after three years of ripening.

To clarify the contribution of flavor components to sample discrimination, 43 differential compounds were identified based on Variable Importance in Projection (VIP) >1 and *p* < 0.05 (Figure 5D) [47]. Further consideration of the flavor activity of VOCs and FAAs (ROAV > 1 and TAV > 1, respectively) led to the final selection of 30 key discriminant markers, comprising 11 VOCs, 3 FAAs, and 16 FFAs (Table 4). These markers displayed distinct group-specific variation patterns across the six ham groups. DLYH1 was distinguished by lauric acid, heptadecanoic acid, and 2,6-dimethylheptan-4-onenone, with the latter contributing fatty and fruity notes. DLYH2 featured 2-methyl-1-pentanol, which contributed grassy, fruity, and alcoholic notes. In DLYH3, 2-heptanone and a higher level of Arg were more prominent, corresponding to fruity aroma and bitter taste, respectively. In contrast, XWH exhibited more pronounced changes in its characteristic compounds during ripening. XWH1 was marked by erucic acid and hexanenitrile, whereas XWH2 showed a distinctive association with 2-methylpyrazine, which contributed nutty and cocoa-like notes. XWH3 contained higher levels of Glu, Gly, linoleic acid, and oleic acid, together with 2,5-dimethylfuran and 2-ethylfuran. Glu and Gly contributed mainly to umami and sweet tastes, respectively, whereas the two furans were associated with roasted and caramel-like aromas. Although 1-octen-3-ol exhibited high ROAVs in all ham samples because of its low odor threshold, its relative aroma contribution was more pronounced in DLYH.

On this basis, a preliminary panel of discriminant markers was proposed for distinguishing XWH from DLYH. The characteristic flavor-contributing compounds of XWH included Glu, 2-methylpyrazine, 2,5-dimethylfuran, oleic acid, and linoleic acid. In contrast, DLYH was primarily characterized by 2-heptanone and Arg. These compositional differences suggest that the raw-material breed influences the formation, accumulation, and transformation of flavor-related compounds during ham ripening. Taken together, and considering the known taste and aroma properties of these differential compounds, XWH exhibited stronger umami-related responses and a broader range of aroma-associated compounds, particularly pyrazines and furans linked to roasted, nutty, cocoa-like, and caramel-like attributes, whereas DLYH showed higher bitterness-related responses and prominent lipid-oxidation-related volatiles exhibiting fruity, grassy, fatty, and mushroom-like attributes.

## 4. Conclusions

This study demonstrated that pig breed significantly influenced the metabolite profiles and flavor characteristics of dry-cured ham during ripening. Compared with XWH produced from Xuanhe pigs, DLYH showed lower umami intensity, higher bitterness, and fewer characteristic pyrazines and furans. In addition, long-term maturation can amplify the flavor differences arising from breed-related variation in the raw materials. The integration of GC-IMS, biomimetic sensory analysis, targeted metabolite profiling, and chemometrics enabled clear discrimination between the two ham types and facilitated the screening of candidate discriminant markers that may aid their differentiation. Higher levels of Glu, 2,5-dimethylfuran, and linoleic acid were associated with authentic XWH, whereas higher levels of Arg and 2-heptanone were more characteristic of DLYH. These findings provide new insights into breed-dependent flavor evolution in traditional dry-cured ham and establish an analytical basis for future XWH authenticity evaluation, quality control, and rapid screening of DLY-derived substitution. They may also support the market protection of XWH as a PGI product and guide standardized production using authentic raw materials. Future studies incorporating larger industrial sample sets, sensory validation, and model flavor recombination of the screened candidate markers will further strengthen the robustness and practical applicability of the breed-discrimination model.

## Figures and Tables

**Table 1:** Basic physicochemical properties of ham samples.

	DLYH1	DLYH2	DLYH3	XWH1	XWH2	XWH3
Moisture content (%)	46.49 ± 0.38 aB	44.79 ± 1.24 bB	40.75 ± 1.39 cA	49.95 ± 1.47 aA	49.52 ± 0.48 aA	41.44 ± 0.71 bA
Water activity (a_w_)	0.84 ± 0.00 aA	0.78 ± 0.03 bB	0.66 ± 0.01 cB	0.87 ± 0.01 aA	0.84 ± 0.00 aA	0.81 ± 0.00 abA
pH	5.99 ± 0.03 aA	5.83 ± 0.02 bA	5.75 ± 0.03 cA	5.87 ± 0.05 aB	5.81 ± 0.04 aA	5.73 ± 0.02 bA
Crude fat (g/100 g)	7.98 ± 0.55 bA	7.95 ± 0.15 bA	8.54 ± 0.71 aB	8.20 ± 0.44 bA	8.37 ± 0.24 bA	9.53 ± 0.54 aA
Crude protein (g/100 g)	32.06 ± 0.32 bB	33.60 ± 1.53 bA	35.60 ± 1.10 aB	33.49 ± 1.11 bA	33.01 ± 0.76 bA	38.81 ± 0.71 aA

Within the same row, different lowercase letters indicate significant differences among ripening times within the same breed, whereas different uppercase letters indicate significant differences between breeds at the same ripening time (*p* < 0.05).

**Table 2:** FAA contents of different ham samples (mg/g).

FAA	DLYH1	DLYH2	DLYH3	XWH1	XWH2	XWH3
Asp	1.500 ± 0.077 cA	1.762 ± 0.073 bB	2.128 ± 0.044 aB	1.229 ± 0.010 cB	2.277 ± 0.108 bA	3.713 ± 0.273 aA
Thr	2.153 ± 0.123 bA	2.456 ± 0.126 aA	2.097 ± 0.050 bB	1.916 ± 0.021 cB	2.372 ± 0.122 bA	3.613 ± 0.185 aA
Ser	1.833 ± 0.085 bA	2.095 ± 0.098 aA	1.941 ± 0.038 bB	1.691 ± 0.016 cB	1.999 ± 0.064 bA	3.278 ± 0.170 aA
Glu	4.319 ± 0.221 cB	5.405 ± 0.170 bA	6.154 ± 0.152 aB	5.118 ± 0.037 cA	5.560 ± 0.148 bA	9.444 ± 0.452 aA
Gly	1.613 ± 0.070 bA	1.917 ± 0.108 aA	1.695 ± 0.039 bB	1.517 ± 0.040 cA	1.758 ± 0.122 bB	2.932 ± 0.117 aA
Ala	3.637 ± 0.166 bA	3.959 ± 0.145 aA	3.925 ± 0.114 aB	2.999 ± 0.027 cB	3.828 ± 0.247 bA	6.185 ± 0.237 aA
Cys	0.045 ± 0.002 bA	0.032 ± 0.003 bA	0.130 ± 0.026 aA	0.022 ± 0.003 bB	0.029 ± 0.001 bA	0.141 ± 0.012 aA
Val	2.589 ± 0.129 bA	2.906 ± 0.092 aA	2.807 ± 0.086 aB	2.157 ± 0.007 bB	2.908 ± 0.157 aA	3.182 ± 0.132 aA
Met	1.319 ± 0.074 aA	1.377 ± 0.045 aA	1.349 ± 0.036 aB	0.926 ± 0.021 cB	1.454 ± 0.125 bA	1.887 ± 0.071 aA
Ile	2.140 ± 0.124 aA	2.275 ± 0.083 bA	3.197 ± 0.084 aA	1.605 ± 0.005 cB	2.285 ± 0.200 bA	3.083 ± 0.134 aA
Leu	3.473 ± 0.174 cA	3.771 ± 0.156 bA	4.694 ± 0.136 aA	2.635 ± 0.012 cB	3.822 ± 0.274 aA	3.521 ± 0.241 bB
Tyr	0.846 ± 0.088 cB	1.346 ± 0.239 aA	1.164 ± 0.081 bA	1.226 ± 0.025 aA	0.824 ± 0.050 cB	1.032 ± 0.063 bA
Phe	2.276 ± 0.164 aA	2.365 ± 0.071 aA	2.240 ± 0.099 aB	1.637 ± 0.019 cB	2.355 ± 0.235 bA	3.148 ± 0.164 aA
Lys	4.368 ± 0.275 bA	5.123 ± 0.206 aA	5.300 ± 0.141 aB	3.795 ± 0.024 cB	4.921 ± 0.242 bA	7.795 ± 0.363 aA
His	1.152 ± 0.075 bA	1.320 ± 0.059 aA	1.292 ± 0.025 aB	1.004 ± 0.006 cB	1.220 ± 0.111 bB	2.019 ± 0.100 aA
Arg	2.712 ± 0.042 bA	2.667 ± 0.175 bA	3.974 ± 0.183 aA	2.178 ± 0.012 cB	2.353 ± 0.289 bB	2.790 ± 0.309 aB
Pro	1.818 ± 0.077 bA	1.806 ± 0.080 bB	1.920 ± 0.060 aB	1.587 ± 0.025 cB	1.940 ± 0.141 bA	2.964 ± 0.107 aA
Umami amino acids	5.829 ± 0.234 cB	7.167 ± 0.185 bB	8.282 ± 0.158 aB	6.347 ± 0.038 cA	7.837 ± 0.183 bA	13.157 ± 0.528 aA
Sweet amino acids	15.422 ± 0.369 cA	17.356 ± 0.327 aA	16.878 ± 0.205 bB	13.505 ± 0.065 cB	16.818 ± 0.416 bA	26.767 ± 0.526 aA
Bitter amino acids	16.507 ± 0.331 cA	18.027 ± 0.371 bA	20.717 ± 0.291 aA	13.368 ± 0.043 cB	17.221 ± 0.556 bB	20.662 ± 0.485 aA
Others	0.045 ± 0.002 bA	0.032 ± 0.003 bA	0.130 ± 0.026 aA	0.022 ± 0.003 bB	0.029 ± 0.001 bA	0.141 ± 0.012 aA
Total	37.793 ± 0.549 cA	42.582 ± 0.528 bA	46.007 ± 0.390 aB	33.242 ± 0.087 cB	41.905 ± 0.718 bA	60.727 ± 0.889 aA

Note: Umami amino acids include Asp and Glu; sweet amino acids include Thr, Ser, Pro, Gly, Lys and Ala; bitter amino acids include Val, Met, Ile, Leu, Phe, His, Tyr and Arg; and other amino acids include Cys [33]. Within the same row, different lowercase letters indicate significant differences among ripening times within the same breed, whereas different uppercase letters indicate significant differences between breeds at the same ripening time (*p* < 0.05).

**Table 3:** FFA contents of different ham samples (g/100 g).

FFA	DLYH1	DLYH2	DLYH3	XWH1	XWH2	XWH3
Hexanoic acid (C6:0)	0.196 ± 0.043 bA	0.180 ± 0.029 bA	0.263 ± 0.053 aA	0.143 ± 0.020 bA	0.169 ± 0.008 abA	0.219 ± 0.029 aA
Decanoic acid (C10:0)	0.021 ± 0.001 aA	0.017 ± 0.003 aA	0.021 ± 0.003 aA	0.012 ± 0.002 bB	0.018 ± 0.001 aA	0.019 ± 0.002 aA
Lauric acid (C12:0)	0.020 ± 0.001 aA	0.013 ± 0.005 bA	0.018 ± 0.005 abA	0.012 ± 0.002 aB	0.011 ± 0.001 aA	0.017 ± 0.003 aA
Myristic acid (C14:0)	0.049 ± 0.003 cA	0.066 ± 0.005 bA	0.085 ± 0.008 aB	0.039 ± 0.003 cB	0.065 ± 0.003 bA	0.094 ± 0.003 aA
Palmitic acid (C16:0)	1.447 ± 0.005 cA	1.984 ± 0.127 bA	3.085 ± 0.091 aA	1.693 ± 0.077 bA	1.945 ± 0.293 bA	3.200 ± 0.215 aA
Palmitoleic acid (C16:1)	0.201 ± 0.000 bA	0.254 ± 0.019 bA	0.344 ± 0.007 aB	0.188 ± 0.006 cA	0.263 ± 0.034 bA	0.495 ± 0.077 aA
Heptadecanoic acid (C17:0)	0.022 ± 0.001 aA	0.015 ± 0.000 bA	0.015 ± 0.003 bA	0.012 ± 0.001 bB	0.010 ± 0.001 bB	0.015 ± 0.001 aA
(Z)-10-Heptadecenoic acid (C17:1)	0.022 ± 0.001 aA	0.016 ± 0.001 bA	0.017 ± 0.003 bA	0.011 ± 0.001 bB	0.011 ± 0.001 bB	0.016 ± 0.001 aA
Stearic acid (C18:0)	0.536 ± 0.003 bB	0.645 ± 0.037 bB	1.110 ± 0.041 aA	0.825 ± 0.056 bA	1.025 ± 0.125 aA	1.122 ± 0.207 aA
Elaidic acid (C18:1n9t)	0.016 ± 0.001 aA	0.015 ± 0.001 aA	0.016 ± 0.003 aA	0.010 ± 0.001 bB	0.008 ± 0.000 bB	0.014 ± 0.001 aA
Oleic acid (C18:1n9c)	2.659 ± 0.161 cB	3.515 ± 0.101 bB	5.066 ± 0.176 aB	3.285 ± 0.086 cA	4.054 ± 0.089 bA	5.469 ± 0.131 aA
Linolelaidic acid (C18:2n6t)	0.012 ± 0.000 bA	0.013 ± 0.001 bA	0.020 ± 0.001 aB	0.011 ± 0.000 bA	0.013 ± 0.002 bA	0.025 ± 0.003 aA
Linoleic acid (C18:2n6c)	1.196 ± 0.012 cB	1.244 ± 0.006 bB	1.540 ± 0.005 aB	1.212 ± 0.004 cA	1.468 ± 0.004 bA	1.952 ± 0.006 aA
α-Linolenic acid (C18:3n3)	0.005 ± 0.000 b	0.006 ± 0.000 aB	ND	ND	0.007 ± 0.000 aA	ND
γ-Linolenic acid (C18:3n6)	0.013 ± 0.002 bB	0.011 ± 0.001 bB	0.017 ± 0.003 aA	0.026 ± 0.001 aA	0.018 ± 0.001 bA	0.019 ± 0.001 bA
Arachidic acid (C20:0)	0.008 ± 0.000 cB	0.015 ± 0.000 bA	0.024 ± 0.000 aA	0.014 ± 0.001 bA	0.013 ± 0.002 bA	0.022 ± 0.004 aA
(Z)-11-Eicosenoic acid (C20:1n9)	0.022 ± 0.003 bB	0.020 ± 0.002 bB	0.035 ± 0.011 aB	0.045 ± 0.004 aA	0.036 ± 0.002 aA	0.045 ± 0.004 aA
(Z,Z)-11,14-Eicosadienoate (C20:2)	0.022 ± 0.002 bB	0.019 ± 0.001 bB	0.028 ± 0.006 aA	0.040 ± 0.002 aA	0.028 ± 0.001 bA	0.031 ± 0.002 bA
Erucic acid (C22:1n9)	0.015 ± 0.001 bB	0.011 ± 0.000 cB	0.018 ± 0.003 aA	0.024 ± 0.001 aA	0.017 ± 0.001 bA	0.019 ± 0.001 bA
Saturated fatty acid (∑SFA)	2.122 ± 0.019 cB	2.785 ± 0.181 bA	4.385 ± 0.156 aA	2.622 ± 0.144 bA	3.104 ± 0.426 bA	4.510 ± 0.437 aA
Monounsaturated fatty acid (∑MUFA)	2.935 ± 0.168 cB	3.830 ± 0.123 bB	5.497 ± 0.202 aB	3.564 ± 0.099 cA	4.390 ± 0.126 bA	6.059 ± 0.216 aA
Polyunsaturated fatty acid (∑PUFA)	1.248 ± 0.017 bA	1.293 ± 0.009 bB	1.606 ± 0.014 aB	1.289 ± 0.008 cA	1.534 ± 0.008 bA	2.027 ± 0.012 aA
Total FFAs (FFA)	6.306 ± 0.204 cB	7.908 ± 0.313 bB	11.487 ± 0.372 aB	7.475 ± 0.251 cA	9.027 ± 0.560 bA	12.596 ± 0.665 aA
UFA/SFA	1.971	1.839	1.620	1.851	1.909	1.793

Note: ‘ND’ indicates not detected. Within the same row, different lowercase letters indicate significant differences among ripening times within the same breed, whereas different uppercase letters indicate significant differences between breeds at the same ripening time (*p* < 0.05).

**Table 4:** Key discriminant compounds in different ham samples.

Compound	Threshold	Flavor Attributes	DLYH1	DLYH2	DLYH3	XWH1	XWH2	XWH3	VIP Value
VOC (ROAV)	µg/kg								
(E)-2-Pentenal	980	Fruity, floral fragrance	0.002	0.002	0.001	0.006	0.005	0.003	1.286
2-Methyl-1-pentanol	0.1	grassy taste, fruity, alcoholic flavor	24.783	30.752	24.012	11.953	10.206	10.060	1.199
Alpha-Pinene	6	Cedar, pine, sharp	0.853	2.967	1.618	2.064	1.821	1.676	1.118
1-Octen-3-ol	1	Mushroom, soil flavor	100.000	100.000	100.000	100.000	100.000	100.000	1.112
2,5-Dimethylfuran	1.8	Baking fragrance, burnt fragrance	1.503	5.141	1.655	2.602	8.755	10.679	1.108
2,6-Dimethyl-4-heptanone	9.3	Slight oily, Fruity	1.627	0.891	0.677	0.675	0.779	0.301	1.106
Furfural	3000	Almonds, baked potatoes, bread, burnt aromas	0.002	0.003	0.001	0.002	0.002	0.002	1.106
1-Propanethiol	3.1	Onion, cabbage flavor	0.496	0.983	0.307	1.590	0.938	0.885	1.098
4-Hexen-1-ol	100	grassy taste	0.077	0.103	0.065	0.142	0.098	0.073	1.090
Cyclopentanone(M)	51.1	mint flavor	0.169	0.424	0.111	0.269	0.207	0.168	1.077
1-Octene	150	Oil flavor	0.049	0.048	0.086	0.126	0.077	0.056	1.076
Acetal	42	Cream, fruity, sweet.	0.007	0.037	0.007	0.052	0.019	0.012	1.071
2-Heptanone	1.3	sweet fruity, mild creamy	1.641	1.992	2.629	1.411	1.272	1.035	1.065
3-Methyl-3-buten-1-ol	550	Green leaf flavor	0.135	0.231	0.112	0.287	0.187	0.171	1.061
2-Ethylfuran	2.3	Butter, caramel	2.211	2.991	1.883	2.375	4.110	6.362	1.053
(E)-2-octen-1-ol	40	Mushroom flavor	0.127	0.212	0.130	0.236	0.159	0.174	1.049
Hexyl butanoate	250	Green, fruity, citrus aromas	0.009	0.025	0.009	0.054	0.014	0.013	1.042
Delta-3-carene	9.3	Lemon fragrance	0.401	1.054	0.549	0.436	0.398	0.516	1.035
2,3-Pentadione	40	sweet aroma, creamy	0.048	0.130	0.203	0.072	0.034	0.144	1.029
1-Nonanol	50	Fat, citrus flavor	0.029	0.089	0.026	0.117	0.037	0.039	1.023
3-Hydroxybutan-2-one(D)	800	Butter, cream	0.006	0.012	0.004	0.008	0.007	0.006	1.019
Hexanenitrile(D)	3.2	grassy, raw green, slight oily	4.244	4.690	6.902	12.974	10.457	10.397	1.016
2-Isobutyl-3-methyl pyrazine	35	Nuts, roasted peanuts, herbaceous aroma	0.011	0.027	0.007	0.157	0.022	0.012	1.010
2-Methylpyrazine	80	Cocoa, nutty, roast scent	0.431	0.627	0.246	1.782	2.747	1.404	1.009
FAA (TAV)	mg/g								
Glu	0.3	umami	14.397	18.017	20.513	17.060	18.533	31.480	1.350
Arg	0.5	bitter	5.424	5.334	7.948	4.356	4.706	5.580	1.069
Gly	1.3	sweet	1.241	1.475	1.304	1.167	1.352	2.255	1.005
FFA (concentration)	g/100 g								
Heptadecanoic acid (C17:0)			0.022	0.015	0.015	0.012	0.010	0.015	1.300
α-Linolenic acid (C18:3n3)			0.005	0.006	ND	ND	0.007	ND	1.222
Linoleic acid (C18:2n6c)			1.196	1.244	1.540	1.212	1.468	1.952	1.199
(Z)-10-Heptadecenoic acid (C17:1)			0.022	0.016	0.017	0.011	0.011	0.016	1.170
Stearic acid (C18:0)			0.536	0.645	1.110	0.825	1.025	1.122	1.130
Lauric acid (C12:0)			0.020	0.013	0.018	0.012	0.011	0.017	1.114
Arachidic acid (C20:0)			0.008	0.015	0.024	0.014	0.013	0.022	1.110
Oleic acid (C18:1n9c)			2.659	3.515	5.066	3.285	4.054	5.469	1.073
Elaidic acid (C18:1n9t)			0.016	0.015	0.016	0.010	0.008	0.014	1.073
Erucic acid (C22:1n9)			0.015	0.011	0.018	0.024	0.017	0.019	1.052
Decanoic acid (C10:0)			0.021	0.017	0.021	0.012	0.018	0.019	1.025
(Z)-11-Eicosenoic acid (C20:1n9)			0.022	0.020	0.035	0.045	0.036	0.045	1.023
Palmitic acid (C16:0)			1.447	1.984	3.085	1.693	1.945	3.200	1.021
(Z,Z)-11, 14-Eicosadienoate (C20:2)			0.022	0.019	0.028	0.040	0.028	0.031	1.017
Myristic acid (C14:0)			0.049	0.066	0.081	0.039	0.065	0.094	1.014
γ-Linolenic acid (C18:3n6)			0.013	0.011	0.017	0.026	0.018	0.019	1.008

Note: ‘ND’ indicates not detected. Odor thresholds were obtained from Compound Threshold Compilation (Second Edition), and odor descriptors were obtained from FlavorNet and FEMA.

## Data Availability

The original contributions presented in this study are included in the article/Supplementary Materials. Further inquiries can be directed to the corresponding author.

## Supplementary Materials

The following supporting information can be downloaded at: https://www.mdpi.com/article/10.3390/foods15203592/s1, Table S1. GC-IMS determination of VOCs in different hams.
