# Supplementary material for: Integrated Flavoromics Reveals Breed-Associated Flavor Evolution in Dry-Cured Xuanwei Ham During Ripening
Source: Foods. 2026 Oct 10;15(20):3592. doi: 10.3390/foods15203592 (PMC13703340; doi:10.3390/foods15203592)
Supplement: Supplementary file 1 [file foods-15-03592-s001.zip › foods-4539373-supplementary.pdf]

Table S1. GC-IMS determination of VOCs in different hams

Compounds	CAS	RI	Rt	Intensity					
				DLYH1	DLYH2	DLYH3	XWH1	XWH2	XWH3
						Alcohols			
3-Methyl-2-butanol	C598754	689.2	140.456	5719.63±68.41[cB]	6285.65±13.76[aA]	6019.09±55.63[bB]	5987.45±46.62[bA]	5520.35±61.08[cB]	6535.57±47.48[aA]
3-Methyl-3-buten-1-ol	C763326	745.7	168.731	8943.86±83.79[bB]	8245.60±82.39[cB]	9805.22±76.91[aA]	10080.95±53.86[aA]	8398.27±41.38[cA]	9746.54±77.19[bA]
2-Butanol	C78922	607.4	109.151	3460.30±36.45[bB]	3870.31±26.54[aB]	3468.71±69.64[bB]	3705.39±95.29[cA]	4206.66±44.76[bA]	4899.17±49.01[aA]
Linalool(M)	C78706	1106.5	610.012	180.54±5.85[bA]	170.98±3.33[cB]	236.97±6.32[aA]	178.78±5.85[bA]	201.87±6.45[aA]	160.39±5.96[cB]
Linalool(D)	C78706	1106.5	610.012	226.35±6.46[bA]	279.19±4.70[aA]	227.34±5.98[bA]	170.47±3.00[cB]	199.08±8.44[bB]	223.93±6.70[aA]
(E)-2-octen-1-ol	C18409171	1070.9	534.149	613.67±5.95[bA]	550.97±8.73[cA]	824.90±7.62[aA]	600.91±9.31[bB]	520.12±5.59[cB]	720.02±5.16[aB]
(E)-3-hexen-1-ol	C928972	832	226.003	389.60±5.10[cB]	446.09±7.77[bB]	522.66±7.26[aA]	477.23±8.04[bA]	576.26±9.10[aA]	408.94±9.21[cB]
4-Methyl-2-pentanol	C108112	748	170.005	2041.30±75.94[bA]	2223.87±38.62[aA]	1848.34±28.68[cA]	1397.33±32.25[bB]	1798.13±16.19[aB]	1779.18±47.59[aB]
2-Ethoxyethanol	C110805	709.8	150.067	196.74±3.03[bA]	240.85±7.70[aB]	241.01±8.87[aB]	161.16±6.07[cB]	248.32±5.35[bA]	351.94±5.48[aA]
2-Hexanol	C626937	814	212.427	249.05±4.31[bA]	159.48±3.37[cB]	300.18±5.47[aB]	107.06±6.30[cB]	216.24±8.66[bA]	356.42±9.71[aA]
2-Methyl-1-pentanol	C105306	832.1	226.033	166.17±7.32[cA]	393.81±5.91[aA]	222.99±6.03[bB]	140.02±2.43[cB]	165.41±7.05[bB]	280.14±6.61[aA]
1-Nonanol	C143088	1177.7	795.452	175.50±3.23[cB]	289.36±6.78[aA]	207.08±8.56[bA]	372.98±8.21[aA]	153.07±5.81[cB]	204.21±5.55[bA]
1-Octanol	C111875	1070.4	533.213	51.84±1.98[cA]	64.56±0.87[bA]	82.55±1.41[aA]	41.10±1.34[cB]	50.50±1.49[bB]	72.18±1.68[aB]
1-Propanethiol	C107039	623.6	114.567	185.27±3.74[bB]	197.57±9.81[aB]	151.66±3.65[cB]	314.30±9.30[aA]	237.95±7.45[cA]	284.16±5.76[bA]
4-Hexen-1-ol	C928927	885.7	272.64	926.39±7.70[bA]	665.83±6.60[cB]	1039.78±11.54[aA]	905.34±10.57[aB]	798.92±9.34[bA]	758.78±10.20[cB]
2-Hexen-1-ol	C928950	863.2	215.903	38.66±1.64[cB]	43.35±2.20[bB]	59.69±1.86[aA]	122.65±3.45[aA]	109.21±6.67[bA]	20.64±1.16[cB]
1-Octen-3-ol	C3391864	974.1	374.289	602.8±1.54[cA]	724.1±1.06[bA]	795.7±2.54[aA]	318.9±0.23[cB]	409.3±1.31[bB]	517.6±1.42[aB]

1-Penten-3-ol	C616251	672.8	133.353	39.41±0.88^{aB}	33.55±0.66^{cB}	36.95±0.90^{bA}	56.10±1.51^{aA}	45.76±0.89^{bA}	34.25±0.64^{cB}
Ketones									
2-Heptanone(M)	C110430	892	278.774	714.89±4.43^{bB}	576.68±10.92^{cB}	913.66±8.23^{aA}	963.35±6.36^{bA}	1065.73±15.12^{aA}	747.07±8.59^{cB}
2-Heptanone(D)	C110430	885.2	272.175	300.51±9.99^{bB}	212.97±8.05^{cB}	554.82±7.06^{aA}	452.36±7.18^{aA}	360.32±6.82^{bA}	229.30±4.82^{cB}
3-Hydroxybutan-2 -one(M)	C513860	731.2	160.881	3891.35±30.12^{aB}	3620.74±57.75^{bB}	3527.01±30.02^{cA}	4553.03±48.90^{aA}	4248.29±39.45^{bA}	3278.39±48.15^{cB}
3-Hydroxybutan-2 -one(D)	C513860	731.2	160.881	548.31±4.92^{bA}	601.10±8.22^{aA}	551.31±3.68^{bA}	421.92±8.24^{cB}	440.42±5.50^{bB}	534.93±6.94^{aB}
2-Pentanone	C107879	689.9	140.771	205.53±8.11^{bA}	223.87±6.00^{aA}	189.05±6.39^{cA}	105.76±5.24^{cB}	151.70±2.58^{bB}	176.00±5.51^{aB}
2-Methyltetrahydrofuran-3 -one	C3188009	813.8	212.266	286.72±5.01^{bA}	916.58±5.67^{aA}	285.93±5.89^{bB}	88.91±3.52^{cB}	463.17±6.19^{bB}	669.8±6.89^{aA}
3,5,5-Trimethyl-2-cyclohexen-1-one	C78591	1145.5	705.542	400.11±6.97^{bA}	368.42±3.71^{cA}	803.09±7.49^{aA}	191.37±4.38^{cB}	228.93±4.67^{bB}	649.53±4.33^{aB}
Cyclopentanone(M)	C120923	781.3	190.015	1042.29±30.76^{bA}	1405.06±24.27^{aA}	903.10±11.78^{cA}	876.30±4.65^{abB}	867.84±2.01^{bB}	886.19±9.97^{aA}
Cyclopentanone(D)	C120923	781.6	190.216	158.93±5.45^{bB}	122.49±4.21^{cB}	402.07±6.94^{aA}	260.02±3.67^{aA}	192.55±3.94^{bA}	150.35±7.11^{cB}
2,6 Dimethyl-4-heptanone	C108838	974.1	374.289	1824.67±21.90^{aA}	537.24±8.47^{cB}	1002.41±13.34^{bA}	400.46±8.70^{bB}	593.14±4.88^{aA}	290.00±6.85^{cB}
2,3-Pentanedione	C600146	666.3	130.693	91.53±5.15^{cB}	224.77±7.80^{bA}	452.28±6.60^{aA}	184.43±3.35^{bA}	102.61±5.41^{cB}	290.79±5.53^{aB}
N-Methylpyrrolidone	C872504	1048.2	491.067	108.19±4.21^{bA}	104.63±7.58^{bA}	289.37±4.22^{aA}	85.39±2.80^{bB}	78.64±2.22^{cB}	191.94±6.39^{aB}
2-Nonanone	C821556	1090.4	574.422	286.62±7.77^{bB}	333.17±9.61^{aA}	231.89±2.49^{cA}	410.74±5.88^{aA}	231.20±5.97^{bB}	120.69±3.86^{cB}
Esters									
Ethyl 2-hydroxy-4-methylpentanoate	C10348477	1069.9	532.276	1007.47±13.40^{bA}	1157.51±35.55^{aA}	953.84±4.83^{cA}	698.41±5.89^{cB}	859.74±2.87^{bB}	922.36±3.16^{aB}
Isoamyl acetate	C123922	864.3	252.819	216.92±8.67^{cB}	355.49±7.92^{aA}	254.40±3.81^{bA}	317.28±9.82^{aA}	250.74±5.26^{bB}	136.61±3.04^{cB}

Isopropyl 2-methylbutanoate	C66576714	875.2	262.711	116.40±6.18^{cB}	151.32±3.13^{bA}	158.43±3.96^{aA}	143.69±4.52^{aA}	117.83±2.73^{bB}	107.46±4.52^{cB}
Isopentyl formate	C110452	781.5	190.175	675.96±5.03^{cA}	777.47±7.11^{aA}	712.86±5.23^{bA}	495.34±7.73^{bB}	432.34±5.29^{cB}	575.48±5.65^{aB}
Propyl propanoate	C106365	810.2	209.642	65.72±2.64^{cA}	97.50±2.11^{bA}	119.96±4.1^{aB}	48.00±1.47^{cB}	57.44±1.46^{bB}	425.96±8.59^{aA}
Acetic acid butyl ester	C637649	1090.4	574.422	382.57±5.96^{bA}	481.92±5.59^{aA}	383.75±7.62^{bB}	127.08±3.08^{cB}	207.26±8.51^{bB}	777.33±8.99^{aA}
Isobutyl propionate	C540421	864.6	253.094	381.73±6.56^{bB}	280.46±7.55^{cB}	574.43±3.16^{aA}	1137.78±51.94^{aA}	676.57±8.02^{bA}	253.78±3.97^{cB}
Ethyl heptanoate	C106309	1109.4	616.568	624.67±6.35^{bB}	720.72±5.01^{aB}	590.82±5.92^{cA}	1289.82±27.16^{aA}	774.96±9.85^{bA}	478.49±8.67^{cB}
Butyl butanoate	C109217	997.8	408.057	586.63±6.36^{bB}	528.03±5.25^{cB}	884.39±9.40^{aA}	1082.75±32.5^{aA}	773.02±8.15^{bA}	480.54±10.79^{cB}
Hexyl butanoate	C2639636	1178	796.389	270.45±6.70^{cB}	397.18±8.47^{aA}	362.54±6.40^{bA}	854.01±6.84^{aA}	286.55±9.67^{cB}	333.06±7.76^{bB}
Allyl heptanoate	C142198	1177.1	793.579	80.78±1.54^{bB}	90.09±2.65^{aA}	90.76±4.41^{aA}	166.86±3.31^{aA}	79.78±3.30^{cB}	90.00±4.25^{bA}
					Aldehydes				
Octanal	C124130	995.7	404.903	958.97±5.95^{bB}	948.13±5.87^{bA}	1088.94±21.91^{aA}	1349.03±30.11^{aA}	949.51±5.39^{bA}	813.83±5.32^{cB}
Heptanal	C111717	892.6	279.335	1760.37±42.44^{bA}	2041.91±27.71^{aA}	1687.52±45.04^{cB}	1736.89±26.68^{bA}	1396.89±43.68^{cB}	1939.88±57.47^{aA}
Furfural	C98011	839.8	232.17	652.78±5.97^{aA}	509.63±11.93^{bB}	450.50±4.93^{cB}	414.00±7.59^{bB}	600.15±8.71^{aA}	604.56±9.21^{aA}
2-Methylpropanal	C78842	772.4	184.427	52.86±0.92^{bA}	67.73±2.67^{aA}	45.16±1.34^{cB}	31.60±1.07^{cB}	37.72±0.41^{bB}	54.37±0.66^{aA}
Butanal	C123728	612.9	110.964	4104.11±46.95^{bA}	4335.57±45.81^{aA}	3921.85±47.46^{cB}	3630.05±25.78^{cB}	3967.36±23.53^{bB}	4347.40±39.23^{aA}
2-Methylpentanal(D)	C123159	775.7	186.494	859.10±3.97^{bA}	892.06±9.24^{aA}	777.12±2.51^{cA}	211.50±9.22^{cB}	482.11±4.31^{bB}	723.92±4.90^{aB}
2-Methylpentanal(M)	C123159	775.7	186.494	639.92±5.39^{bA}	1044.48±20.01^{aA}	336.97±4.59^{cB}	185.70±6.33^{bB}	555.26±9.21^{aB}	552.50±8.67^{aA}
(E)-2-Pentenal	C1576870	758.3	175.945	212.56±8.35^{bB}	251.38±6.51^{aB}	202.73±8.65^{cB}	363.09±5.07^{bA}	406.77±8.24^{aA}	315.51±6.55^{cA}
2-Methyl-2-pentenal	C14250965	829.6	224.128	86.26±2.22^{cB}	138.47±1.84^{bB}	213.79±4.85^{aA}	384.70±8.07^{aA}	263.24±7.86^{bA}	184.41±7.64^{cB}
Acetal	C105577	712.5	151.383	37.73±2.00^{cB}	101.71±4.62^{aA}	46.05±1.24^{bB}	138.39±5.07^{aA}	64.23±1.72^{bB}	52.64±1.09^{cA}

				Acids					
2-Methylpropanoic acid	C79312	772.5	184.502	839.52±10.95^{cB}	931.96±21.51^{bB}	1201.84±16.09^{aB}	891.36±6.84^{cA}	1730.59±62.26^{bA}	2856.63±45.77^{aA}
				Hydrocarbon					
2,2,4,6,6-pentamethylheptane	C13475826	985.1	389.5	562.43±8.33^{bA}	519.21±5.40^{cB}	628.26±6.64^{aA}	528.40±3.27^{bB}	545.93±7.68^{aA}	362.59±3.17^{cB}
				Terpenes					
Delta-3-carene	C13466789	1012.8	431.127	449.94±6.67^{cA}	635.16±8.59^{bA}	712.82±9.66^{aA}	258.61±2.81^{cB}	303.02±6.60^{bB}	496.58±8.11^{aB}
Allyl cyanide	C109751	655.2	126.217	852.10±5.74^{cB}	1185.43±29.80^{aA}	1139.75±20.35^{bA}	955.34±7.86^{bA}	855.65±8.74^{cB}	1128.66±20.02^{aA}
Alpha-Pinene	C80568	939.2	329.977	616.98±25.54^{cB}	1153.87±30.53^{bA}	1544.69±37.54^{aA}	789.91±10.87^{cA}	894.51±13.15^{bB}	1041.10±24.76^{aB}
1-Octene	C111660	781.1	189.901	891.83±10.33^{bB}	464.70±10.23^{cB}	2051.42±90.52^{aA}	1204.56±15.33^{aA}	941.06±18.47^{bA}	867.57±17.36^{cB}
Terpinolene	C586629	1092.2	578.168	185.32±4.11^{aB}	171.15±5.91^{bB}	120.39±2.66^{cA}	305.12±5.28^{aA}	203.53±6.48^{bA}	97.00±2.00^{cB}
Beta-Myrcene	C123353	995	403.966	112.92±4.67^{bB}	81.31±3.10^{cB}	250.85±6.28^{aA}	342.8±9.47^{aA}	149.19±7.42^{bA}	82.25±2.68^{cB}
				Others					
N,N-diethylethanamine	C121448	709.3	149.835	106.42±1.87^{cA}	146.69±4.51^{aA}	139.91±5.60^{bA}	57.79±1.53^{cB}	121.98±2.21^{bB}	137.85±4.33^{aA}
2,5-Dimethylfuran	C625865	706.4	148.437	326.18±7.33^{cA}	599.86±8.09^{aB}	473.93±8.08^{bB}	298.79±9.58^{cA}	1290.17±35.48^{bA}	1989.97±55.33^{aA}
2-Ethyl furan	C3208160	700.6	145.673	829.48±8.55^{aA}	744.13±9.36^{bB}	689.29±5.90^{cB}	348.44±7.46^{cB}	773.96±9.70^{bA}	1514.84±29.11^{aA}
2-Methylthiophene	C554143	766.7	180.927	83.64±1.76^{aA}	77.78±1.48^{bB}	63.69±1.15^{cB}	69.60±2.06^{cB}	99.25±2.37^{bA}	139.7±3.55^{aA}
Pyrrole	C109977	774.6	185.815	143.02±5.39^{aB}	89.49±6.21^{bA}	91.69±5.47^{bA}	157.41±5.29^{aA}	79.21±3.08^{bB}	70.03±3.65^{cB}
2-Methylpyrazine	C109080	814.9	213.067	4159.09±93.84^{aB}	3253.96±49.42^{bB}	3125.68±50.49^{cB}	9094.60±30.70^{bA}	17989.55±130.67^{aA}	3343.34±82.44^{cA}

Hexanenitrile(M)	C628739	882	269.123	315.77±3.66^{bB}	320.39±6.05^{bB}	403.96±9.01^{aA}	397.99±8.46^{bA}	447.12±4.30^{aA}	310.68±8.46^{cB}
Hexanenitrile(D)	C628739	886.5	273.384	1637.42±53.90^{bB}	972.94±43.02^{cB}	3514.67±62.52^{aA}	4280.93±29.38^{aA}	2739.35±51.40^{bA}	1456.64±36.71^{cB}
γ-Valerolactone	C108292	941.6	332.787	52.24±2.41^{bA}	40.19±2.86^{cB}	180.74±5.25^{aB}	38.35±1.73^{cB}	60.99±2.39^{bA}	215.26±4.54^{aA}
2-Pentylfuran	C3777693	986.3	391.325	202.28±8.67^{bB}	134.89±4.17^{cB}	260.93±3.17^{aA}	218.26±3.89^{aA}	187.34±8.16^{bA}	151.15±4.13^{cB}
2-Isobutyl-3-methyl pyrazine	C13925069	1107.8	612.821	48.41±2.21^{bB}	60.18±2.52^{aA}	41.08±2.14^{cA}	351.54±7.30^{aA}	62.60±0.96^{bA}	44.29±0.67^{cA}
Dipropyl disulfide	C629196	1108.2	613.758	912.14±8.08^{bB}	1055.48±24.34^{aA}	687.03±14.81^{cB}	1361.72±45.43^{aA}	805.97±5.13^{bB}	734.02±23.73^{cA}

Note：The values are presented by means ± standard deviations. Within the same row, different lowercase letters indicate significant differences among ripening times within the same breed, whereas different uppercase letters indicate significant differences between breeds at the same ripening time ($p < 0.05$). n = 6.
